# Carbonized Tree‐Like Furry Magnolia Fruit‐Based Evaporator Replicating the Feat of Plant Transpiration

**DOI:** 10.1002/gch2.201900040

**Published:** 2019-09-13

**Authors:** Yue Bian, Yang Shen, Kun Tang, Qianqian Du, Licai Hao, Dongyang Liu, Jinggang Hao, Dong Zhou, Xiaokun Wang, Huiling Zhang, Peiye Li, Yimeng Sang, Xiu Yuan, Lijuan Zhao, Jiandong Ye, Bin Liu, Hai Lu, Yi Yang, Rong Zhang, Youdou Zheng, Xiang Xiong, Shulin Gu

**Affiliations:** ^1^ National Laboratory of Solid State Microstructures and Collaborative Innovation Center of Solid‐State Lighting and Energy‐Saving Electronics School of Electronic Science and Engineering Nanjing University Nanjing 210093 China; ^2^ School of the Environment Nanjing University Nanjing 210093 China; ^3^ Nanjing Foreign Language School Nanjing 210093 China; ^4^ School of Physics Nanjing University Nanjing 210093 China

**Keywords:** magnolia fruits, thermal management, tree‐like evaporators, water purification

## Abstract

It has long been an aspirational goal to create artificial evaporators that allow omnidirectional energy absorptance, adequate water supply, and fast vapor transportation, replicating the feat of plant transpiration, to solve the global water crisis. This work reveals that magnolia fruits, as a kind of tree‐like living organism, can be outstanding 3D tree‐like evaporators through a simple carbonization process. The arterial pumping, branched diffusion, and confined evaporation are achieved by the “trunk,” “branches,” and “leaves,” respectively, of the mini tree. The mini tree possesses omnidirectional high light absorptance with minimized heat loss and gains energy from the environment. Water confined in the fruit possesses reduced vaporization enthalpy and transports quickly following the Murray's law. A record‐high vapor generation rate of 1.22 kg m^−2^ h^−1^ in dark and 3.15 kg m^−2^ h^−1^ under 1 sun illumination is achieved under the assistance of the gully‐like furry surface. The “absorption of nutrients” enables the fruit to recover valuable heavy metals as well as to produce clean water from wastewater efficiently. These findings not only reveal the hidden talent of magnolia fruits as cheap materials for vapor generation but also inspire future development of high‐performance, full‐time, and all‐weather vapor generation and water treatment devices.

## 1 Introduction

Given the global water and energy crisis, solar‐driven evaporation has become a renewed topic owing to its high conversion efficiency of solar energy and transformative industrial potential.^1^, ^2^, ^3^ Over the last 6 years, in order to improve the solar‐vapor efficiency, numerous artificial 2D evaporation devices have been designed with enhanced light absorptance, confined heat, unremitted water supply, and fast vapor transportation.^4^, ^5^, ^6^, ^7^, ^8^, ^9^, ^10^, ^11^, ^12^, ^13^, ^14^ However, due to reflection (2–5%) and radiation loss occurring in all the 2D evaporators, the conversion efficiency (less than 90%) can hardly be further improved.

The advancement from the 2D structures to the 3D ones is a big step forward. In a typical 3D evaporator, within a given projection area, the actual surface area could be substantially enlarged, resulting in a decrease of the absorber surface temperature.^15^ In this case, the environmental thermal energy could be further utilized. Recently, some unprecedented vaporizing rates have been reported in a variety of the 3D evaporators.^16^, ^17^, ^18^

Herein, we report that the tree‐like furry magnolia fruits, as a natural multibranched and hierarchical material, can be outstanding 3D mini‐tree like evaporators through a simple carbonization process. Such carbonized magnolia fruit‐based 3D artificial transpiration device possesses the following features: 1) natural hydrophilicity; 2) numerous multibranched microchannels that act as highways for fast water transport; 3) gully‐like surface trapping light effectively in a broad wavelength range; 4) tree‐like 3D structure enabling omnidirectional high light absorptance and not suffering the inevitable cosine losses of 2D evaporator; 5) furry surface increasing the evaporation area; 6) staggered pod‐like evaporation unit providing additional available free space for fast vapor escape; 7) rapid thermal response that reduces evaporation rate dependence on the bulk water volume and the detrimental factors by the intermittent nature of solar irradiation (such as low‐insolation periods due to partial cloud cover); 8) reduced heat convection and radiation loss; 9) average temperature that lower than environment; 10) reduced evaporation enthalpy of the water in the magnolia fruit mesh; 11) ability of recycling valuable heavy metal day and night with minimal carbon footprint and producing purified water; 12) good mechanical properties and outstanding corrosion stability; 13) good scalability and readily available that is low cost. Benefiting from the above advantages, a floating carbonized magnolia fruit (CMF) evaporates water with a very high vapor generation rate of 1.22 kg m^−2^ h^−1^ in dark and 3.15 kg m^−2^ h^−1^ under 1 sun. The outdoor experiment shows that the performance of the 3D tree‐like evaporator is 3.7 × that of a traditional 2D evaporator in a typical cold winter day. Moreover, it shows superior stability and reusability for wastewater treatment. No performance degradation occurs after cycling of 240 h. The carbonized magnolia fruits show excellent overall performance as compared to other reported generators, and have attractive applications in wastewater treatment of industry. In the following section, we shall elucidate all of these features.

## 2 Results and Discussion

**Figure 1:**
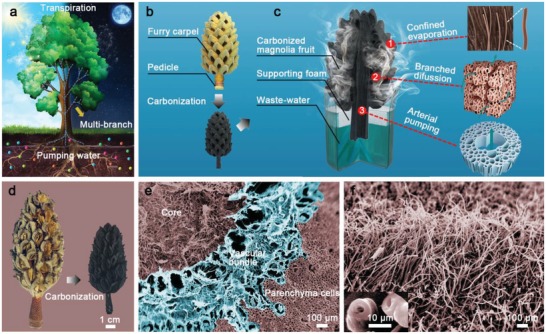
Carbonized magnolia fruit‐based 3D evaporation assisted wastewater treatment. a) Trees transport water up from the bottom and absorb mineral nutrients needed for growth, day and night with the help of leaf transpiration. b) The mature tree‐like furry magnolia fruit undergoes a controllable carbonization process to form the 3D CMF. c) Schematic of the CMF‐based 3D evaporation assisted wastewater treatment device. 1) The vapor generation from the magnolia fruit mesh is enhanced under illumination. 2) Fast water replenishment is escorted by branched diffusion and 3) arterial pumping, respectively, by parenchyma cells and vascular bundles. d) Optical image of a magnolia fruit before and after carbonization. e) SEM micrograph of the transverse section of the CMF's pedicle showing the porous vascular bundles and parenchyma cells. f) SEM micrograph of the surface of the CMF's carpel which showing the tightly arranged plant trichomes. Inset is an SEM micrograph of the cut‐away section of the plant trichomes showing a hollow structure.

Plant transpiration, one of the nature's masterpieces, provides a perfect solution for efficient vapor generation (**Figure** **1**a). In the transpiration process, water is absorbed from the soil, and transported to the roots, stems, and leaves, pursing multibranching while with narrow pores to realize maximum mineral nutrients and water supply.^19^, ^20^ This hierarchically multibranching porous structure is naturally optimized with minimized transport resistance and follows the Murray's law, which is also commonly observed in biological tissues, such as the cardiovascular system.^21^ Inspired by the transpiration and the 3D morphology of the plants, we have demonstrated that the tree‐like magnolia fruits can be excellent 3D artificial transpiration devices. Figure 1b,c shows the illustration of the design concept. Magnolia fruit, a woody fruit made up of numerous furry pod‐like carpels and a porous pedicle, was carbonized to make it black (Figure 1b). The 3D carbonized magnolia fruit (CMF)‐based evaporator was inserted into a piece of polystyrene foam to make it float. Combined with the magnolia fruit's unique multibranched porous structure, a 3D hierarchical and interpenetrating network involving branches and arteries was established, providing channels ideal for water supply. Under the synergy of the capillary force and the natural hydrophilicity of the fruit fiber, the CMF pumps water actively through its inner network and generates clean vapor for freshwater.

Figure 1d presents the woody carcass of the natural magnolia fruit before and after carbonization, looking like a miniature Christmas tree with a stout axis and bushy branches (details in the Experimental Section and Figure S1 in the Supporting Information). The volume of the magnolia fruit shrinks to ≈70% after the carbonization process. However, observation by the scanning electron microcopy (SEM) demonstrates that the microstructure of the magnolia fruit is retained. Figure 1e reveals the highly porous microstructure of the fruit pedicle. The porous and fibrous structures of the pedicle and carpel (details in Figures S2 and S3a in the Supporting Information) provide ideal channels for supplying water. Impressively, the trichomes on the furry carpel surface are well preserved (Figure 1f). The trichomes, hollow tubes having a diameter of about 10 µm and a length of several hundred microns to a few millimeters, increase the evaporation area effectively, just as the leaves act in the transpiration processes of plants (Figure 1a; Figure S3b–d, Supporting Information). The furry carpel possesses a large porosity (0.5097 cm^3^ g^−1^) with uniform pore size of ≈3.9 nm (Figure S4, Supporting Information) and the BET surface area (1050.4 m^2^ g^−1^) is among the highest of recently reported evaporators (Table S1, Supporting Information).

**Figure 2:**
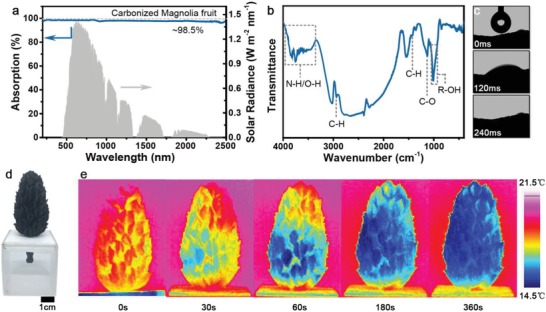
Absorptance and hydrophilicity of the carbonized magnolia fruit. a) Absorption spectra of the 3D CMF. b) FTIR spectrum of the CMF shows the existence of polar groups that facilitate water absorption. c) A water droplet (3 µL) permeates through the CMF rapidly. d) Photograph of a floating CMF used to demonstrate the hydrophilicity. e) IR images of the dry CMF before (0 s) and after (30, 60, 180, 360 s) touching with water.

The X‐ray diffraction (XRD) pattern of the CMF shows a typical amorphous carbon structure and the Raman spectra reveal a higher content of amorphous carbon than graphitized carbon (Figure S5, Supporting Information). The solar absorptance of the 3D CMF is 98.5% (**Figure** **2**a). The CMF traps light effectively as light inside the staggered carpels can undergo increased light–matter interaction length and absorption times. Obviously, as the sky position of the sun is continuously changing, trees instinctively grow as tall and wide as possible to absorb more sunlight. Thanks to the tree‐like 3D structure, the CMF also shows high omnidirectional absorption of light (Figure S6, Supporting Information), and performs better light absorption than 2D devices in real‐world applications (≈470% improvement, Figure S7, Supporting Information).

Besides the efficient light absorptance, hydrophilicity is another essential indicator for an ideal evaporator. The magnolia fruit retains its hydrophilicity after the carbonization process because its constituents are mainly cellulose. Fourier transform infrared (FTIR) spectroscopy showed a typical cellulose structure (Figure 2b).^22^ Plenty of oxygen‐containing functional groups ensuring its hydrophilic nature for water absorption. Impressively, when a water drop is dropped on the CMF's surface, the droplet permeates through the CMF within 240 ms (Figure 2c and Video S1, Supporting Information). As a comparison, it takes several seconds for a flame‐treated wood to absorb a water droplet,^23^ and 20 s for a plasmonic wood,^24^ demonstrating the superior hydrophilicity of the CMF.

The superior hydrophilicity of the CMF leads to high‐efficient water absorption and transportation when it floats on the water (Figure 2d). An infrared camera was employed to monitor the wetting process of the CMF without illumination (Figure 2e). Due to arterial pumping, only ≈30 s is needed for a magnolia fruit to pump water from bottom to top (7.2 cm in height). The cross‐transportation of water is also evident owing to branched diffusion, just like the water‐absorbing process of real trees. By measuring the amount of water absorbed by a dry CMF in 360 s, the water supply capacity was calculated as ≈0.015 kg m^−2^ s^−1^, guaranteeing the water supply for solar vapor generation even under 15‐fold concentrated sun illumination.

**Figure 3:**
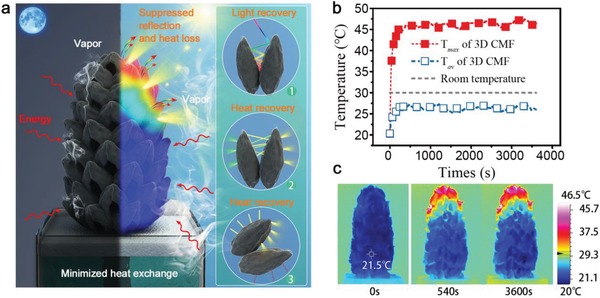
Heat behaviors of the carbonized magnolia fruit. a) Schematic diagram of the CMF's heat behaviors both in the dark and under 1 sun illumination. The 3D CMF possesses suppressed reflection and heat loss by 1) light recovery and 2,3) heat recovery, minimized heat exchange with bulk water, and gains energy from the environment. b) Temperature changes of the 3D CMF over time under 1 sun. c) Infrared photos of the 3D CMF in wet state in dark (left image), with light illumination for 540 s (middle image) and for 3600 s (right image).

As mentioned initially, the tree‐like 3D geometry of the magnolia fruits is naturally optimized for minimizing the heat loss. The heat behaviors of the floating 3D tree‐like CMF‐based evaporator both in dark and under 1 sun illumination have been systematically evaluated by an infrared camera (**Figure** **3**a). Figure 3b plots the maximum temperature (*T*_max_) and the average temperature (*T*_av_) as a function of the illumination duration. Once the wet 3D CMF is exposed to light, *T*_max_ and *T*_av_ increase exponentially. The temperature of the wet CMF in the initial stage is 21.5 °C, 8.5 °C lower than the room temperature (30 °C) due to the strong intrinsic evaporation, corresponding to ≈2488 J h^−1^ energy gain from the environment (the convection contribution is ≈62.7% and the rest by radiation). According to the increasing trend in temperature, *T*_max_ reaches the steady‐state temperature of ≈46.0 °C within ≈540 s, indicating the excellent photothermal properties of the CMF. And *T*_av_ reaches ≈26.3 °C within ≈300 s, 3.7 °C lower than the environment, which is intuitive in infrared pictures (Figure 3c). We note that, as the tilt angle of the top surface is relatively small compared to the side (Figure S8, Supporting Information), the top surface temperature is slightly higher than the ambience.

Another noteworthy phenomenon is that, due to the unique 3D structure of CMF (large in the middle and small at both ends), the bottom of the 3D CMF has similar temperature under dark and 1 sun illumination (Figure S9c, Supporting Information), indicating that heat exchange with the bottom bulk water is minimized. The perfect thermal isolation of the evaporator to the bulk water reduces the evaporation rate dependence on the bulk water volume, leading to a rapid thermal response of the evaporator. This feature is beneficial to improving the device performance under intermittent solar irradiation, as will be shown later.

The average temperature below room temperature is a necessary condition for a net energy gain from the environment. The heat behavior is carefully analyzed (Figure S9, Supporting Information). Quantitatively, the energy input from the sun is 4523.8 J h^−1^ (in a 4 cm diameter circular projection surface) with 67.8 J h^−1^ loss by reflection, and the energy loss from the hotter top area is ≈381.8 J h^−1^ (149.9 and 231.9 J h^−1^ by radiation and convection, respectively). Note that the energy loss from the irradiated surfaces can be partly recovered and recycled by neighboring carpels by multiple energy reflection (close‐up of Figure 3a,2), the concave of the pod‐like carpel and adjacent low temperature surface (close‐up of Figure 3a,3, and Figure S10, Supporting Information), the solar‐to‐vapor conversion efficiency should be higher than 90.1% (calculated efficiency value when considering no energy recovery). At the same time, the cold side surface gains energy from the ambient (≈1775.1 J h^−1^), much higher than the energy loss from the top surface. The net energy input is calculated to be ≈5849.2 J h^−1^, so the 3D CMF could generate vapor beyond the solar energy limit.

The solar‐to‐vapor conversion efficiency (η) can be defined as $\eta =\dot{m}h_{\text{VCMF}}\text{/}P_{\text{in}}$, where *P*_in_ is the 1 sun illumination power (1 kW m^−2^), $\dot{m}$ is the evaporation rate, and *h*_VCMF_ is the evaporation enthalpy of the water confined in CMF.^25^ By the differential scanning calorimetry experiments (Figure S11, Supporting Information), we have confirmed that *h*_VCMF_ is reduced compared to that of bulk water. Therefore, for the same η and *P*_in_, the $\dot{m}$ could be much higher. The reduced *h*_VCMF_ has also been found in other literatures, like in hierarchically nanostructured gel, porous cellulose membrane, and carbonized bamboos. The reduction is possibly attributed to the cluster escape mode of water molecules.^26^, ^27^, ^28^, ^29^

**Figure 4:**
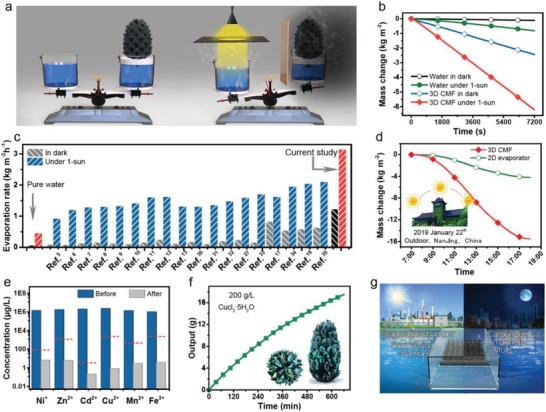
Evaporation performance and two pathways of wastewater treatment using 3D carbonized magnolia fruits. a) Comparison of the evaporation rate of pure water under 1 sun and that of 3D CMF in dark. b) Mass change of water and c) vapor generation rate of 3D CMF in dark and under 1 sun (1 kW m^−2^), with b) pure water and c) previous reported evaporators as the control. d) Mass of water recorded over time outdoors using 3D CMF and 2D Chinese ink‐stained wood. e) Concentrations of various metal ions before and after purification. The WHO standards for drinkable water are indicated by the pink dotted line. f) The output of fresh water over illumination time. Inset shows the photos of the 3D CMF after 11 h illumination and the recovered CuCl_2_·5H_2_O crystal. g) Schematic of using carbonized magnolia fruits for large‐dimension two pathways of wastewater treatment.

All the above results indicate that the CMF is a masterpiece of nature that can be used for excellent 3D artificial transpiration. Impressively, the evaporation rate of the CMF in dark condition is faster even than that of pure water under 1 sun illumination, which can be observed by naked eye when placing them at both ends of the balance (**Figure** **4**a; Video S2, Supporting Information). Specifically, in 80 min, the water evaporated by the CMF in dark is 1 g more than that of pure water (Figure S14, Supporting Information).

To evaluate the vapor‐generation performance of the CMF systematically, the mass change was recorded (Figure 4b). Due to the hugely enlarged surface area (under synergy of macroscopic 3D tree‐like structure and microscopic hairy surface), additional available free space for fast vapor escape (provided by staggered pod‐like carpel), and reduced vaporization enthalpy, the dark evaporation rate of the 3D CMF (1.222 kg m^−2^ h^−1^) is ≈22.5 × that of pure water in dark (0.054 kg m^−2^ h^−1^) and0.766 kg m^−2^ h^−1^ faster than pure water under 1 sun illumination (0.456 kg m^−2^ h^−1^). Meanwhile, the 3D CMF possesses a high evaporation rate of 3.15 kg m^−2^ h^−1^ under 1 sun illumination, corresponding to 5949 J h^−1^ energy input, which accords with the total energy input estimated from the above‐mentioned analysis of the heat behavior. As some parts of the CMF were not illuminated by the sun light, energy input from the environment in these parts should be subtracted to conservatively estimate the solar evaporation rate. The solar evaporation rate is calculated to be 2.28 kg m^−2^ h^−1^ (Figure S9, Supporting Information). The performance of the 3D CMF was compared to the recently reported evaporators (Figure 4c).^30^, ^31^, ^32^, ^33^, ^34^, ^35^ Obviously, the 3D CMF shows excellent evaporation rates both in dark and under illumination. What's more, the evaporation rate shows no dependence on water quantity (Figure S15, Supporting Information) and responses fast to the irradiation change (Figure S16, Supporting Information).

As mentioned initially, the tree‐like 3D CMFs can collect more sunlight throughout the day than horizontal 2D evaporators, as the problem of cosine losses is circumvented. The better performance in real‐world applications is also proved. The solar vapor‐generation experiment was conducted outdoors from 7:00 a.m. to 6:00 p.m. (details in Figure S17, Supporting Information). The total evaporation of the 3D CMF (15.6 kg m^−2^) is 3.7× that of the 2D evaporator (4.2 kg m^−2^) when occupying the same ground area (Figure 4d).

The environmental friendly performance of the 3D CMF involves two aspects: recovering valuable heavy metals as well as producing clean water directly from wastewater efficiently. As a demonstration, various heavy metal ion solutions (Ni^+^, Zn^2+^, Cd^+^, Cu^2+^, Mn^2+^, Fe^3+^; 5000 mg L^−1^) were used to evaluate the vapor quality. After purification, the ion concentrations were all dramatically reduced and the condensate was pure enough to meet the drinking water standards defined by WHO (Figure 4e).^36^ Note that the original ion concentrations are three orders higher than the WHO standards. And the decontamination effect shows no dependent on pH values (Figure S18, Supporting Information).

To carefully check the effect of heavy metal recovery, CuCl_2_, a common pollutant found in wastewater from electroplating industry, was chosen as an example. As shown in Figure 4f, after 11 h of solar irradiation, the CuCl_2_ is precipitated on the 3D CMF's surface. Note that the ion concentration of the actual plating wastewater (0.02–0.1 g L^−1^) is at least 2000 times lower than the current experiment (200 g L^−1^), so the evaporator does not need to be replaced frequently (expected life time of ≈623 days when the original concentration is 0.05 g L^−1^ and the output is 44.7 L m^−2^ d^−1^).

The durability of the CMF‐based evaporator for wastewater treatment was also studied. No performance degradation occurs after cycling of 240 h (Figure S19, Supporting Information). The CMF also exhibits good mechanical properties with a typical compressive stress of 12 MPa (Figure S20, Supporting Information). Furthermore, no obvious deformation was observed even after immersion in a strong acid and alkali solution for 2 months, demonstrating its outstanding corrosion stability (Figure S21, Supporting Information).

Based on the advantages stated above, a prototype for large‐area vapor generation using the 3D tree‐like furry CMFs is proposed (Figure 4g). Transparent plastic can be used to construct the evaporation and condensing chambers. The CMFs are neatly distributed in the evaporation chamber and float on the water. The chamber is sealed during the day, enabling fast clean water production, and is opened during the night, allowing fast heavy metal recovery. Note that, with the rapid growth of vapor generation rate, the collection of condensate has been the Achilles's heel of solar distillation (Figure S22, Supporting Information). It is reported that reducing the pressure of evaporation chamber^37^ or separating the evaporation chamber and condensation chamber in space^38^, ^39^ favor high efficient vapor collection.

## 3 Conclusion

In conclusion, we have shown that magnolia fruits, a widely available biomass waste, by a simple carbonization process, can enable efficient vapor generation day and night. The 3D tree‐like profile possesses omnidirectional energy absorptance, the fibrous Murray evaporator provides continuous water supply, the gully‐like furry surface escorts fast vapor escape and the water confined in the fruit mesh possesses reduced latent heat. Such magnolia fruit‐based high performance, anticorrosive, solid durable, low‐cost, and scalable 3D evaporator is attractive for effective water treatment and recycling of valuable heavy metals, and provides inspiration for future development of advanced 3D designs for high‐performance transpiration devices.

## 4 Experimental Section

*Materials Preparation*: Fresh fruits of evergreen yulan (Magnolia grandiflora) were picked up in the flowerbed at the Nanjing University Xian Lin campus. These magnolia fruits were rinsed by water and dried in air before the carbonization process. The carbonized magnolia fruits were fabricated in a N_2_ atmosphere by a simple two‐step carbonization process. The dry magnolia fruits were carbonized at 350 °C for 1 h followed by a calcination process which was conducted at 900 °C for 5 h.

*Materials Characterizations*: Scanning electron microscopy (EM30, COXEM, Korea) was used to characterize the morphologies of the magnolias. D/MAX‐Ultima III diffractometer was used for the XRD analysis. The absorbance of the carbonized magnolia fruit was measured using a UV/vis/near infrared (NIR) spectrophotometer (Lambda 950, PerkinElmer) with an integrating sphere. FTIR spectra were obtained on a VERTEX70 spectrometer. The surface wettability was evaluated by a contact angle test system (OCA 30, Dataphysics Instruments GmbH, Germany). The infrared photographs were taken by an infrared camera (UTi160b, Uni‐Trend Technology, China). An Instron 5944 instrument was used for the mechanical strain–stress experiments.

*Vapor Generation and Wastewater Treatment*: A carbonized magnolia fruit was inserted into a piece of polystyrene foam with the thermal conductivity of ≈0.03–0.05 W m^−1^ k^−1^, and the whole structure floated on the water surface with only the pedicle in contact with water. The solar spectrum was simulated by a solar simulator (CEL‐S500) with an optical filter for the standard photo radiometer AM 1.5 spectrum. An electronic balance (CP214, 0.1 mg accuracy) was used to record the weight change of water. A serial communication module (RS‐232) was used to connect the electronic balance to the desktop computer. A photoradiometer (CEL‐NP2000) was used to calibrate the illumination density. NiCl·6H_2_O, Zn(CH_3_COO)_2_, CdCl_2_·5H_2_O, CuCl_2_, MnCl_2_·4H_2_O, and FeCl_3_·6H_2_O were used as model metal ion (Ni+, Zn^2+^, Cd^2+^, Cu^2+^, Mn^2+^, Fe^3+^) contaminants to evaluate the wastewater treatment performance. The whole vapor generation experiments were conducted at a humidity of ≈40% and ambient temperature of 30 °C.

## Conflict of Interest

The authors declare no conflict of interest.

## Supporting information

Supplementary

Supplementary

Supplementary
