# Supplementary material for: Carbonized Tree‐Like Furry Magnolia Fruit‐Based Evaporator Replicating the Feat of Plant Transpiration
Source: Glob Chall. 2019 Sep 13;3(10):1900040. doi: 10.1002/gch2.201900040 (PMC6777214; doi:10.1002/gch2.201900040)
Supplement: Supplementary file 1 — Supplementary [file GCH2-3-1900040-s002.pdf]

Copyright WILEY-VCH Verlag GmbH & Co. KGaA, 69469 Weinheim, Germany, 2019.

Supporting Information

for *Global Challenges,* DOI: 10.1002/gch2.201900040

Carbonized Tree-Like Furry Magnolia Fruit-Based Evaporator Replicating the Feat of Plant Transpiration

Yue Bian, Yang Shen, Kun Tang, Qianqian Du, Licai Hao, Dongyang Liu, Jinggang Hao, Dong Zhou, Xiaokun Wang, Huiling Zhang, Peiye Li, Yimeng Sang, Xiu Yuan, Lijuan Zhao, Jiandong Ye, Bin Liu, Hai Lu, Yi Yang, Rong Zhang, Youdou Zheng, Xiang Xiong,* and Shulin Gu**

Supporting Information

Carbonized tree-like furry magnolia fruits-based evaporator replicating the feat of plant transpiration

Yue Bian, Yang Shen, Kun Tang, Qianqian Du, Licai Hao, Dongyang Liu, Jinggang Hao, Dong Zhou, Xiaokun Wang, Huiling Zhang, Yimeng Sang, Xiu Yuan, Lijuan Zhao, Jiandong Ye, Bin Liu, Hai Lu, Yi Yang, Rong Zhang, Youdou Zheng, Xiang Xiong* and Shulin Gu**

S1 Growth morphologies of Magnolia fruit in different periods.

Magnolia is one of the most primitive of all flowering plants, the magnolia's flowering structure hasn't changed significantly in millions of years. As shown in **Figure S1**a, the magnolia bud is pale gray-green and densely hairy (pubescent). Magnolias produce fruit that apparently resembles a cone (pine cone shape). The "cone" of the magnolia isn't really a cone at all, but a woody fruit. During their development, the seeds are contained within the fruit, but as the fruit matures it splits, exposing the gem-like, red seeds.[1]

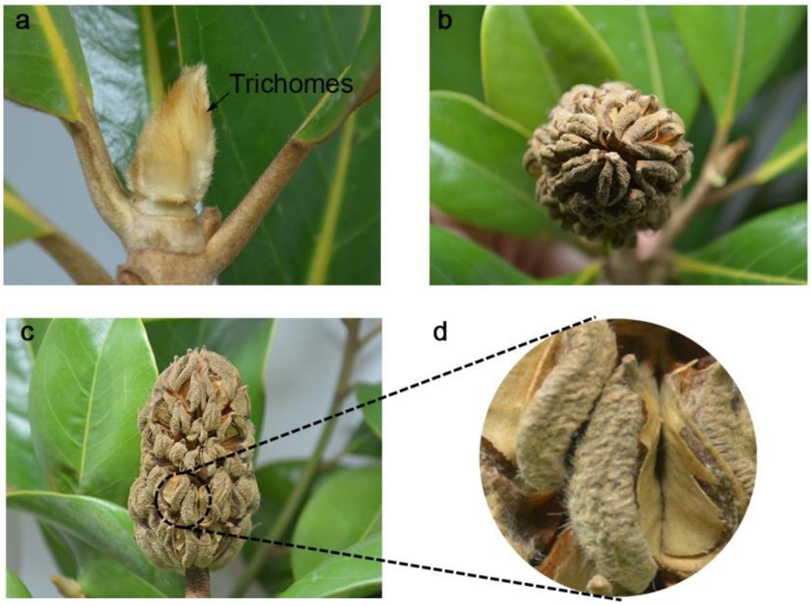

Figure S1. a) Digital photograph of the hairy magnolia buds. b, c) Overlooking (b) and facing (c) photos of a typical mature magnolia fruit. d) Close-up of the mature magnolia fruit, showing the hairy surface.

S2 SEM images of the CMF's pedicle.

As shown in **Figure S2**a, the pedicle comprises two fundamental tissues named vascular bundles (light blue) and parenchyma cells (pink). Within the vascular bundles, the longitudinal tissues that support the whole fruit, the pores (widths ranging from several microns to ~200 microns) are to transport water and nutrients throughout the fruit. The parenchyma cells are woody porous supportive tissue with structure of large holes (tens micrometers) nesting small holes (several micrometers), looking like the famous Chinese puzzle balls (Figure S2 b, c and d).

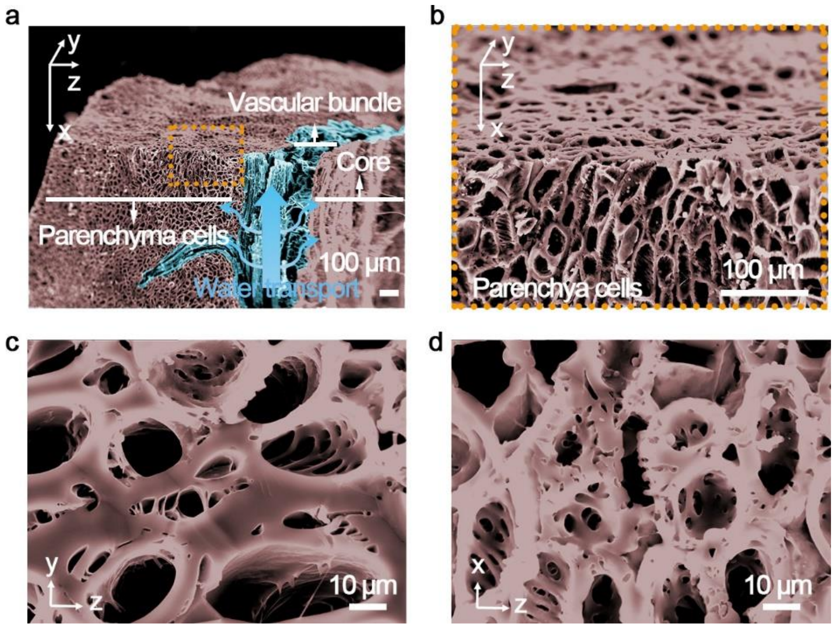

Figure S2. a) SEM micrographs of the CMF's pedicle showing the x (the growth), y and z directions. b) Magnified images of the area indicated in a showing the porous parenchyma cells. c, d) Magnified images of b showing the porous y-z plane (c) and (d) x-z plane.

S3 SEM images of the CMF's carpel.

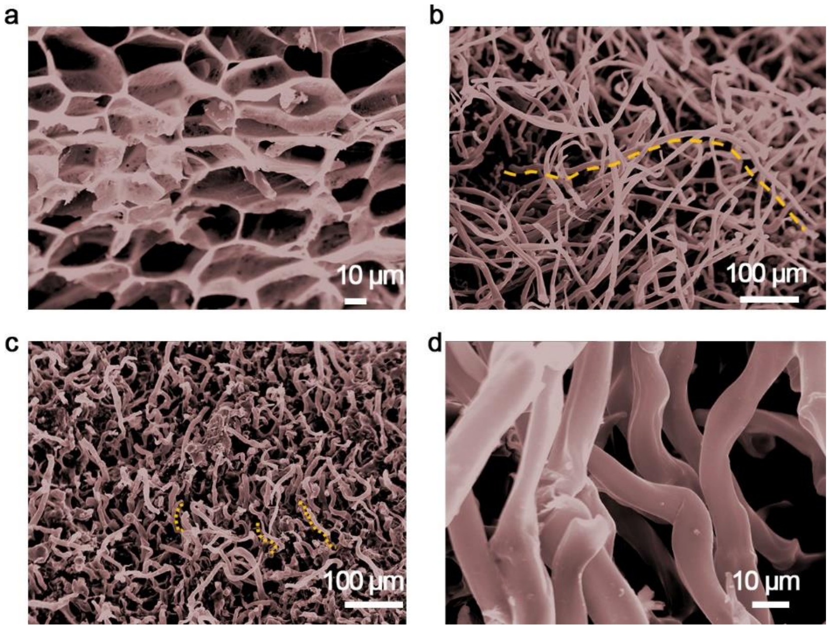

Figure S3. a) SEM micrographs of the CMF's carpel, showing the internal porous structure. b, c) SEM micrographs of the hairy surface of carpel, showing the trichomes ranging from several hundred microns (c) to (b) a few millimeters. d) Magnified images of the trichomes.

S4 Brunauer-Emmett-Teller (BET) test of the CMF

TriStar 3000 gas adsorption analyzer (Micromeritics, USA) was used for the specific surface area measurement of the CMF. The furry carpel possesses a large BET surface (1050.4 m^2 g^{-1}) and porosity (0.5097 cm^3 g^{-1}) with uniform pore size of ≈3.9 nm (Figure S4).

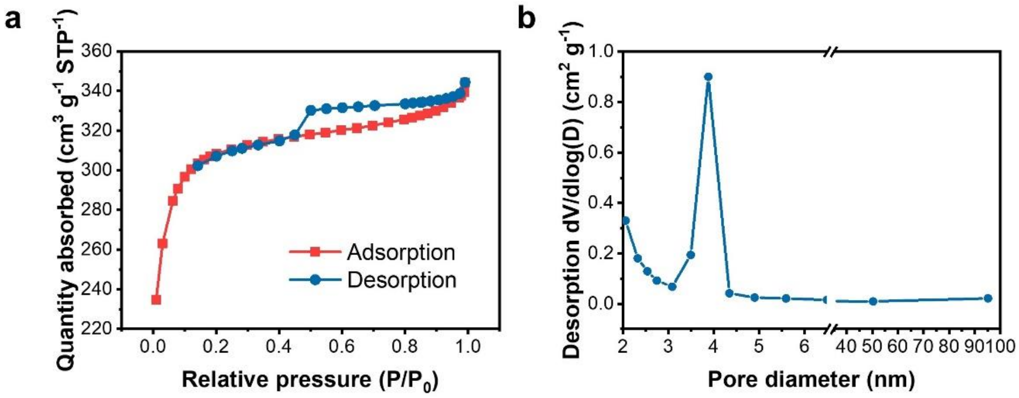

Figure S4. a) The N2 adsorption-desorption isotherm and b) the corresponding Barrett-Joyner-Halenda (BJH) pore size distributions of the furry carpel of the CMF.

This mesoscopic network is favorable for capillary flow, enabling excellent water supply in the solar vapor generation. As a higher surface area can provide more accessible area for vapor generation, so the BET surface area of the CMF has been compared with recently reported evaporators (Supplementary Table 1). Obviously, the surface area of the CMF is among the highest of various evaporators. In addition, it should be noted that, the staggered pod-like carpels also favors vapor escape in space relative to widely adopted 2D evaporators.

Supplementary Table 1. BET surface area of various solar evaporators.

**Ref.**	**Materials**	**BET surface area (m^2 g^{-1})**	**Structure of evaporator**
20	3D graphene	218	2D
21	Mesoporous carbon	467	2D
22	copper-zinc-tin-selenide	11	2D
23	hierarchically porous carbon membrane	499	2D
24	Silica Gel	809	2D
25	MoN_x nanorambutans	23.3	2D
26	hierarchical porous carbon membrane	117.9	2D
27	rGO sponge	119.22	2D
28	PAAm-radial aerogel	4.91	3D
29	crGO	515	2D
30	Graphene Aerogel	44.75	2D
31	bacterial nanocellulose Aerogel	75	2D
32	Carbon from zeolite beta	1704	2D
33	Porous graphene sheets	1260	2D
34	Graphene aerogel	44.85	2D
35	three-dimensional graphene network	1203	2D
36	MoS_2-loaded carbon foam	322.7	2D
37	Fe-doped SiO_2 aerogel	550	2D
38	conjugated microporous polymer carbon aerogels	826	2D
39	carbon foams	36.67	2D
40	chitosan/ZnO scaffold	52.4	2D
41	black titania nanocages	255	2D
42	TCW	206.898	2D
**This work**	**CMF**	**1050.4**	**3D**

S5 XRD and Raman spectrum of the CMF.

The XRD pattern (**Figure S5a**) of the CMF shows two weak and broad diffractions at 23° and 44°, corresponding to typical reflection of graphite and the turbostatic stacking of hexagonal layers of carbon atoms, respectively.[2,3]

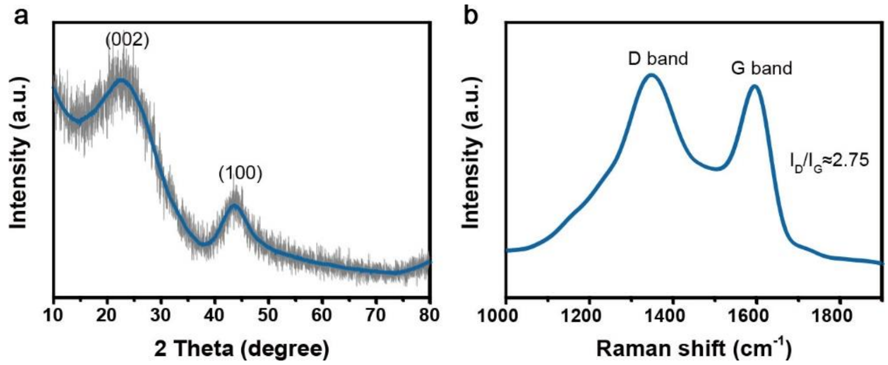

Figure S5. XRD (a) and Raman spectrum (b) of the CMF.

S6 Omnidirectional High Light Antireflection of the 3D CMF

The reflectance characteristics of the 3D CMF was investigated using angle-resolved reflectance spectra (R1 angle-resolved spectroscopy system, Idea Optics, China) and shown in **Figure S6**. The scheme of the measurement is illustrated in Figure S6a. Figure S6b shows the reflection spectrum of a silver mirror, showing that the intensity of the light source is around 32000. Impressively, the 3D CMF show a low reflection with no angle dependence, which is similar to the dark reference. That is the reflection of the 3D CMF is below the limit of detection (0.5%), indicating the omnidirectional high light absorptance of the tree-like 3D CMF.

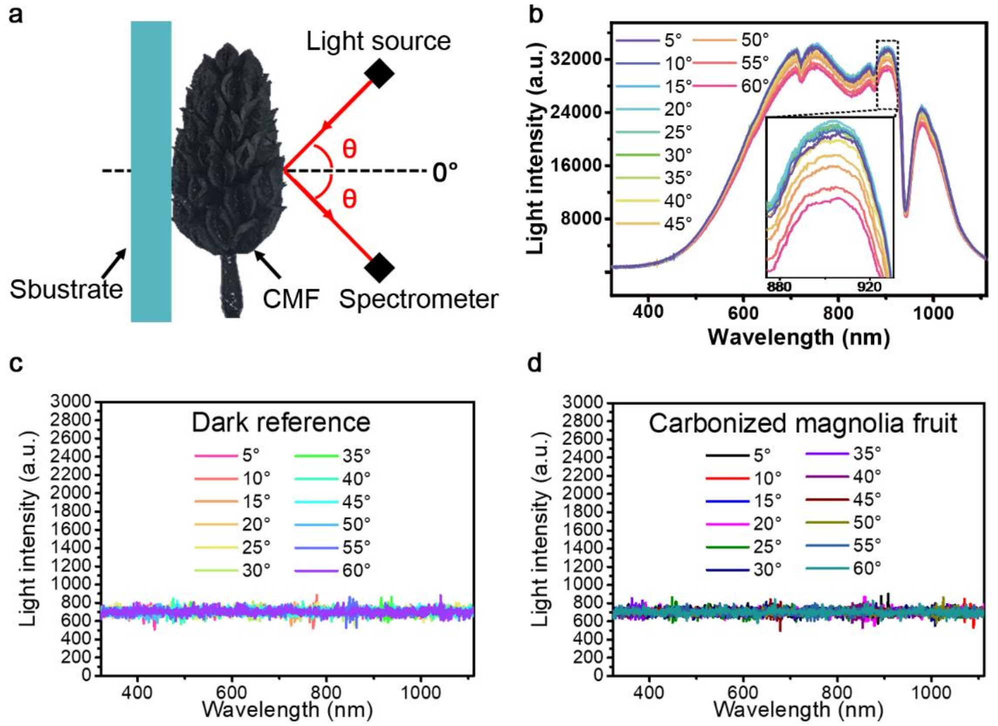

Figure S6. a) Schematic illustration of the angle-resolved reflection spectrum measurement. b) The angle-resolved reflection spectra of a silver mirror showing the spectra of the light source. c, d) The angle-resolved reflection spectra of the dark reference (c) and (d) carbonized magnolia fruit.

S7 Advantages of the Tree-like CMF-based 3D Artificial Transpiration Device.

In this calculation, the latitude is 40°N at 21st March when the sun is above the equator and the length of the day and night is equal all around the world. The proportion of scattered light is 15%.[4-5]

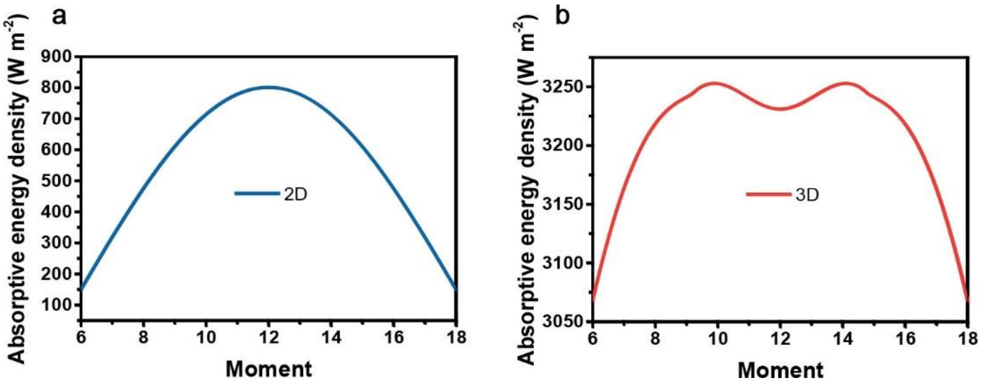

Figure S7. The absorptive amount of 2D plane evaporator (a) and 3D CMF-based artificial transpiration device (b).

S8 Photographs of the 3D CMF.

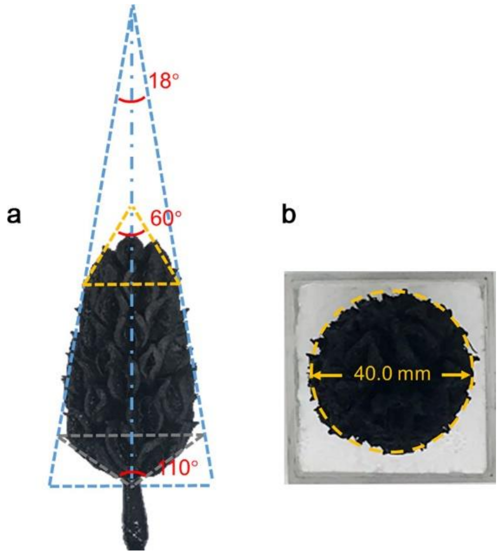

Figure S8. a) Photograph of a typical Christmas tree-like carbonized magnolia fruit, showing a flatter upper surface than the side. b) Physical picture of a carbonized magnolia fruit and the fitting circle for the projection area calculation.

S9 Steady-state Energy Balance Analysis.

The net evaporation rate $\dot{m}$ in a typical solar vapor generation system can be described as

$$\dot{m}h_V = A_{projection}\alpha q_{solar} - A_{surface}\varepsilon\sigma(T_{evaporator}^4 - T_{environment}^4) - A_{surface}h(T_{evaporator} - T_{environment}) - A_{contact}q_{water} \qquad (1)$$

where h_V is the vaporization enthalpy of water in the evaporator, $A_{projection}$ the surface area of the evaporator facing the sun, α the solar absorptance, q_{solar} the solar flux (i.e., 1 kW m^{-2} for 1 sun at AM 1.5), $A_{surface}$ the surface area of the evaporator, ε the emittance of the absorbing surface, σ the Stefan-Boltzmann constant (i.e., 5.67×10^{-8} W m^{-2} K^{-4}), T the surface temperature of the absorber, $T_{environment}$ the temperature of the adjacent environment, h the

convection heat transfer coefficient, $A_{contact}$ the contact area between the absorber and the water surface, and q_{water} the heat flux to the underlying water including conduction and radiation.[6] The second and the third item on the right side of Eq. (1) correspond to radiative and convective heat loss to the ambient, respectively, and the fourth item is the heat loss to the underlying water.

Obviously, when the evaporator temperature $T_{evaporator}$ is lower than the room temperature $T_{environmet}$, the evaporator gains energy from the environment by convection and radiation, vice versa.

Under dark conditions (**Figure S9a**), the energy consumed by natural evaporation is in balance with the energy gains from convection and radiation from the warmer environment, conductive heat gain from the underlying bulk water to the magnolia, and other (if any). We note that the conductive heat gain from bulk water is neglected as the contact area $A_{contact}$ (diameter of the magnolia fruit root: ~6 mm) between the magnolia fruit and underlying water is quite small.

(1) Radiation energy gain $P_{radiation}$:

The radiative heat flux from environment to the CMF is based on Stefan-Boltzmann's law, which is calculated as follows:

$$P_{radiation} = \iint_D^0 \varepsilon\sigma[T_{environment}^4 - T_{(x,y)}^4]d_x d_y \quad (1)$$

(2) Convection energy gain $P_{convection}$:

The convection energy gain is based on Newton's law, which is calculated as follows:

$$P_{convection} = \iint_D^0 h[T_{environment} - T_{(x,y)}]d_x d_y \quad (2)$$

where D refers to the surface of the magnolia fruit and $T_{(x,y)}$ the surface temperature.

Under 1-sun illumination (Figure S9b), the temperature of CMF's top surface is higher than the environment while the rest section is lower than the environment. The energy loss and gain can be calculated using eq.1 and eq.2.

Supplementary Table 2 summarizes the energy input and loss channels of the 3D CMF based evaporator.

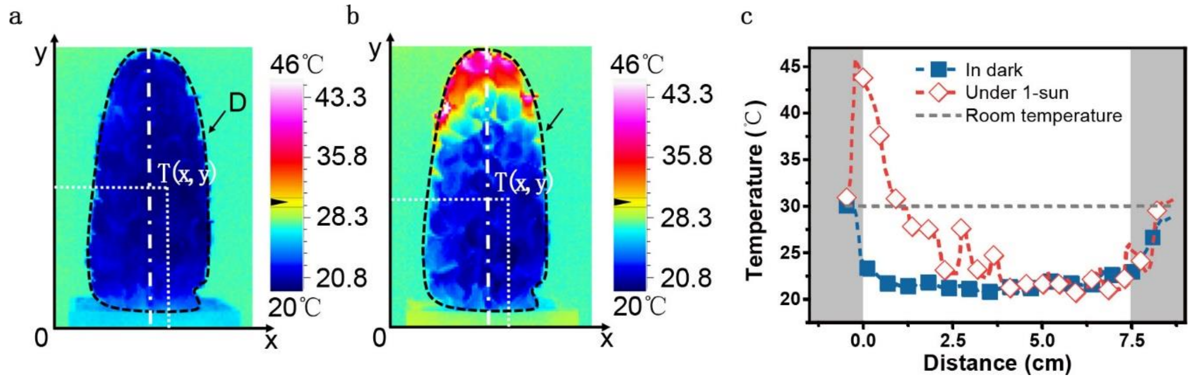

Figure S9. a, b) Infrared photos of the carbonized magnolia fruit in wet state in dark (a) and under 1-sun illumination for 3600 s (b). c) Temperature profiles of the marked dash dots in (a) and (b). The top of the magnolia fruit is set as the zero point of height.

Supplementary Table 2. Summary of energy gain and loss channels in the CMF based 3D evaporator.

	Light input ($J\ h^{-1}$)	Radiation ($J\ h^{-1}$)	Convection ($J\ h^{-1}$)	Net energy input ($J\ h^{-1}$)
In dark	0	gain: 928.2	gain: 1560.0	2488.2
Under 1-sun	4455.9	loss: 149.9	loss: 231.9	
		gain: 664.3	gain: 1110.8	5849.2

According to the steady-state energy balance analysis (S9, Supporting Information), when the CMF-based evaporator is under 1-sun illumination, the energy gain from the environment of the cold surface is 1775.1 J h^{-1} (664.3 J h^{-1} and 1110.8 J h^{-1} by radiation and convection, respectively). So the dark evaporation is 1775.1 J h^{-1}×(1.22 kg m^{-2} h^{-1}/2488 J h^{-1})=0.87 kg m^{-2} h^{-1} , where 1.22 kg m^{-2} h^{-1} and 2488 J h^{-1} are the vapor generation rate and the corresponding energy gain from the warmer environment when the evaporator is under dark. Thus, the solar evaporation rate is 3.15 kg m^{-2} h^{-1}-0.87 kg m^{-2} h^{-1}=2.28 kg m^{-2} h^{-1}.

S10 Energy Recovery and Recycling Pathways on the Hot Top Surface.

A close-up infrared photo of the CMF's top surface is shown in **Figure S10a**. We note that the dissipated heat via radiation from the hot top surfaces can be partly recovered and recycled by the valley between neighboring carpels (red arrows), the concave of the pod-like carpel (blue arrow), and adjacent low temperature surface (Figure S10b). The spatial temperature distributions were investigated to obtain more insight into the energy recovery and recycle process (Figure S10c and d). As shown in Figure S10c, in the horizontal direction, the valleys of temperature were observed in valleys between neighboring carpels (red arrows) and the concave of the pod-like carpel (blue arrow), where less light can arrive. This spatial temperature distribution would lead to a directional heat flow inside the CMF from the warmer part to the colder valleys and concaves, indicating by the red arrows in Figure S10c. In the vertical direction, the heat loss from the hot up surface can be absorbed by the adjacent low temperature surface, which is clearly verified from the temperature gradient (Figure S10d). According to Planck's law, a blackbody at around 40 °C radiates photons at near 10 μm.[7] The excellent mid-infrared absorptivity of the carbon materials enables a highly efficient reabsorption process in self-radiated thermal waves.[8,9]

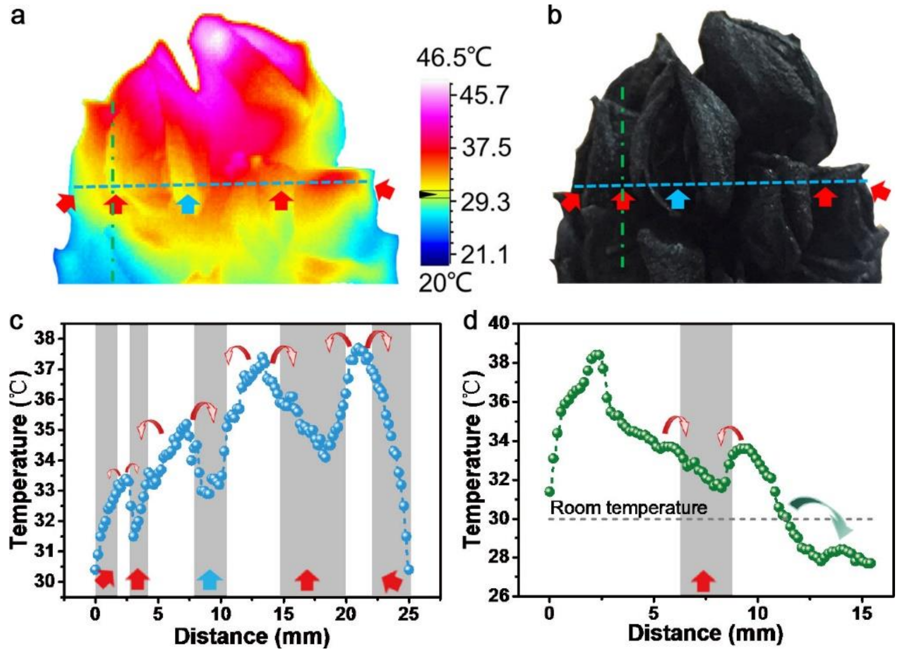

Figure S10. a, b) Close-up infrared photo (a) and (b) corresponding physical picture of the CMF's top surface. c, d) Horizontal (c) and vertical (d) spatial temperature distributions on the creased surface of the tree-like 3D CMF evaporator. The curved arrows indicate the direction of energy dissipation.

S11 DSC Measurement of Vaporization Enthalpy.

Differential scanning calorimetric (DSC) measurement is used for measuring the vaporization energy of pure water and water in magnolia fruit. Measurements have been carried out by exploring the evaporation process of water from 30 to 200 °C for scan rate 5 K min^{-1}, under nitrogen flow flux (20 mL/min). During the vaporization enthalpy measurements, the water and wet carbonized magnolia fruit were placed in a Al crucible with a perforated lidding piece.

For comparison purpose, the changes of heat flow signal of pure water as a function of temperature were also recorded (**Figure S11**a). For pure water, a distinct well-defined peak was observed and the heat flow signal decreased dramatically immediately after the signal reaches maximum, due to the exhaustion of water evaporation process. This indicates that the evaporation process of water is completed immediately. For water in magnolia fruit, the peak is much broader than that of the pure water because water evaporation is not yet completed right after the peak, and the delayed evaporation of the remaining bound water leads to a broader decline of the curve. This indicates that the water evaporation in magnolia fruit is different from pure water.

The vaporization enthalpy is calculated by integrating the heat flux in the time range from 0 to 20 min, with a straight line chosen as the base line (Figure S11b). Supplementary Table 3 summarizes the total mass, mass of water and the calculated vaporization enthalpy of pure water and water in magnolia fruit. The measured enthalpy of water is 2450 J/g, which is very close to the theoretical value of 2444 J/g,[10] indicating the accuracy of our measurements.

The vaporization enthalpy of the water in magnolia fruit is much smaller than that of pure water, due to the influence of the magnolia fruit network on the evaporation process.

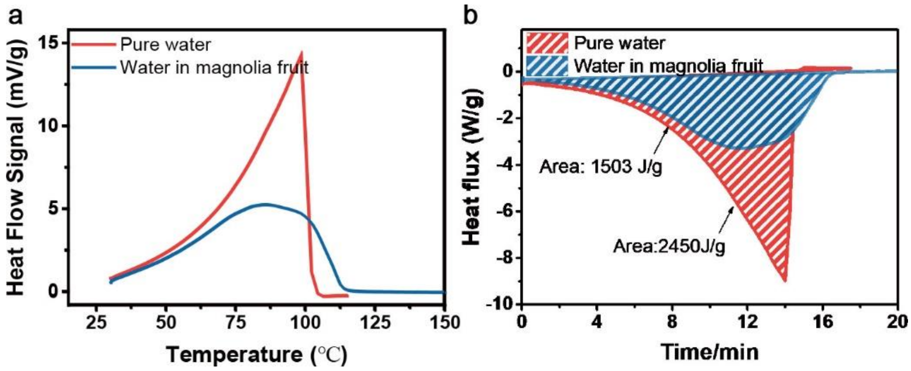

Figure S11. (a) Thermograms of water in magnolia fruit and pure water, the magnitudes of the DSC signal were proportional to the heat flow during the measurements. (b) DSC test curves showing the relationship between heat flux as a function of time.

Supplementary Table 3. Summary of DSC measurement results.

	Water in CMF	Pure water
Total mass (mg)	5.080	9.248
Water (mg)	3.130	9.248
Enthalpy (J/g)	1503	2450

S12 Magnolia Fruit Mesh Enabled Activation of Water

When water is confined within nanoscale space, resulting from the influence of water/wall interaction, its structure and thermodynamic and dynamic properties are very different from those of bulk water.[12] Water molecules confined in the magnolia fruit mesh are more likely to escape the space as small clusters, which consisted of a few to tens molecules, rather than individual molecules. Therefore, a lower energy demand of solar vapor generation is achieved.

To prove the existence of different escape types of water molecules, we compared the vibrational dynamics of interfacial water confined in magnolia fruit (Fig. S12c) with that of bulk pure water (Fig. S12b) by Raman measurements. **Figure S12a** shows the assignments of

five-Gaussian components of OH stretching Raman bands. The three low frequency side components with the center wavenumbers at 3050, 3223 and 3393 cm^{-1} are assigned to hydrogen-bonded water; the band at 3506 cm^{-1} to weakly hydrogen bonded water molecules (whose hydrogen bonds have been broken in part); and the band at 3624 cm^{-1} to free water molecules (whose hydrogen bonds have been broken entirely).[13, 14] The hydrogen-bonded water whose center wavenumber locate at 3050 cm^{-1} is defined as bonding water (BW), the bands at 3223 cm^{-1} and 3393 cm-1 are defined as free water (FW), while the remaining two high frequency side components (whose center wavenumber locate at 3506 and 3624 cm^{-1}) are defined as intermediate water (IW). It is reported that the evaporation rate of IW was ca. 86 times faster than FW.[15] Therefore, the water evaporation enthalpy can be reduced if there could be more IW as they are more evaporable.

When water is confined in CMF, the water molecules captured by nearby fruit mesh through strong interaction, such as hydrogen bonding, are nonfreezable water, the so called BW. The water molecules far from the fruit mesh exhibit the same properties with those in bulk water, so they are FW. The water molecules in the intermediate region between BW and FW interplay delicately with the fruit mesh and adjacent water molecules, and they are IW. The ratios of bound water in bulk water and water confined in CMF mesh are 2.99% and 7.65%, respectively (Figure S12d). This result indicates that water molecules are captured by the magnolia fruit mesh when they are confined in CMF mesh. The calculated molar ratio of IW/FW in water and water confined in CMF mesh are 29.2% and 56.7%, respectively (Figure S12d). Hydrogen bonds weakening resulting from surface tension leads to the relatively high ratio of intermediate water in pure water.[16] This result confirms the proportion of intermediate water increases when water is confirmed in the magnolia fruit mesh, resulting in reduced latent heat and facilitating the water evaporation.

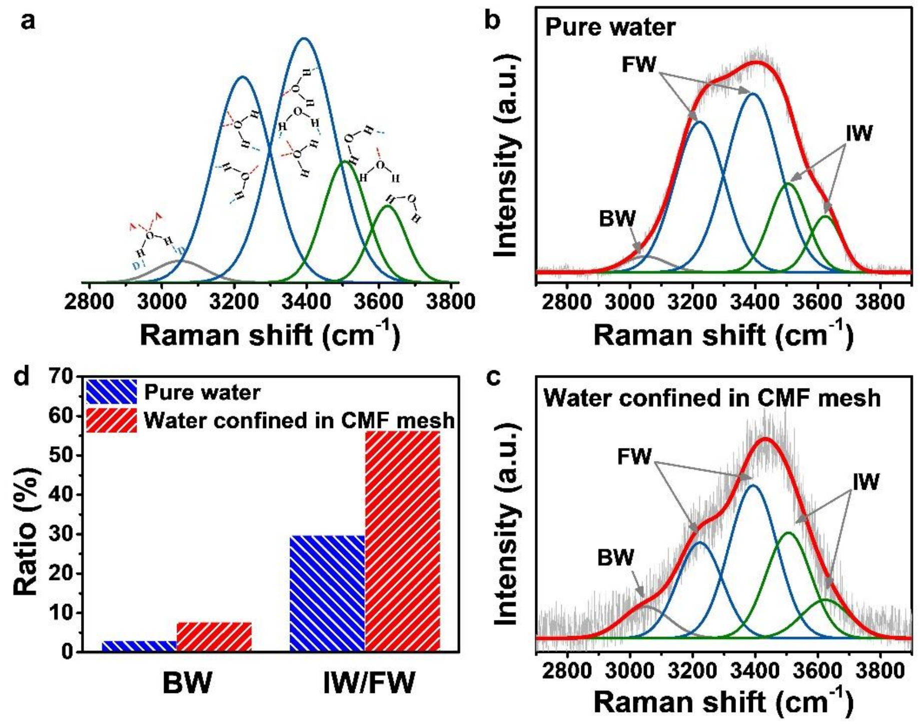

Figure S12. Assignments of five-Gaussian components of OH stretching Raman bands (a) and Raman spectra of OH stretching of (b) pure water and (c) pure water confined in magnolia fruit mesh. Hydrogen bonds are drawn with dashed line. Sky-blue capital letter D and red capital letter A are noted as donor and acceptor of proton, respectively. d) Ratios of BW and IW/FW in pure water and water confined in CMF mesh.

S13 Carbonized Magnolia Fruit Driven Evaporation of Water Clusters

It is reported that the mean velocity of vapor molecules increases with the decreasing size of water cluster, suggesting that it takes lower energy for a monomer to escape from water clusters compared with bulk water.[17] Lithium chloride is a nonvolatile electrolyte and can be loaded by water clusters.[18, 19] We therefore infer that when the water was evaporated by carbonized magnolia fruit (water-cluster evaporation), the Li^+ can be carried to the condensate by water cluster in the vapor (Fig. S13a) and the concentration of Li^+ in the condensate should be higher than that by the traditional evaporation (Fig. S13b).

To experimentally demonstrate that such latent heat reduction was due to evaporation of water clusters from the magnolia fruit mesh confined water, LiCl solutions were used for the solar steam generation experiment, and the concentration of Li^+ in the condensate, evaporated by carbonized magnolia fruit and traditional evaporation, was tested by inductively coupled plasma mass spectrometry (ICP-MS). As shown in Figure S13c, the concentration of Li^+ in the condensate from CMF-based evaporator presented a dependence on the concentration of LiCl solution, while the condensate from traditional evaporation showed a low concentration of Li^+ with no dependence on the Li^+ concentration of the original solution. These results, in addition to the DSC measurement, confirmed our hypothesis that water evaporated from CB-based evaporator is more likely to be in clusters.

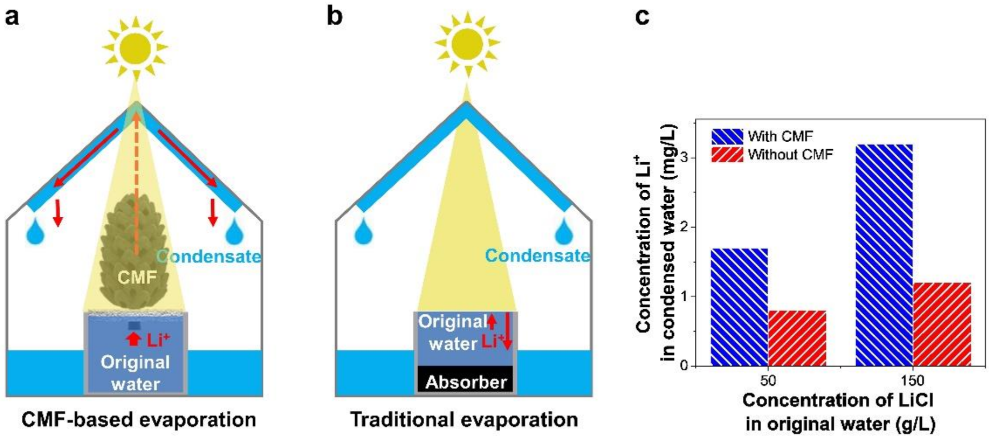

Figure S13. The experimental setup of (a) CMF-based evaporation and (b) traditional evaporation. c) The concentration of Li^+ in condensate from different LiCl solutions.

S14 Comparison of Evaporation Rates of 3D CMF Under Dark and Pure Water Under 1-sun Illumination.

The evaporation rates of 3D CMF under dark and pure water under 1-sun illumination was compared. As shown in **Figure S14**a, the floating 3D CMF and pure water with the same projection area were put on the right and left of the balance, respectively. Evidently, as the illumination time increases, the balance tilts to the side of the pure water, demonstrating that

the evaporation rate of CMF in dark unilluminated condition is faster even than that of pure water under 1-sun illumination (Figure S14 b-e).

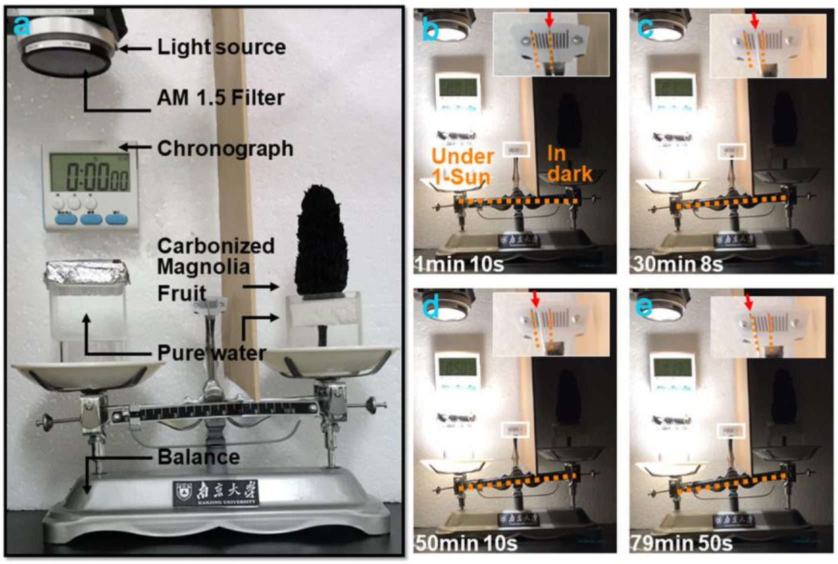

Figure S14. a) The photograph of the experiment setup. b-e) The photographs of the balance showing the tilting process.

S15 The Performance of Devices with Different Water Quantity

Since heat exchange with bulk water in the 3D CMF-based evaporator is minimized, a stable water evaporation rate can be achieved with no dependence on water quantity. For comparison, the dependence of evaporation rates on water quantity for a 2D evaporator with direct bulk water contact and the 3D CMF was carefully examined. Here, Chinese ink-stained ginkgo wood was chosen as a representative of the 2D evaporator, as the ginkgo wood possesses a porous structure (**Figure S15**a) and Chinese ink is a powerful photothermal material.[11] The solar absorptance (weighted by the AM 1.5G solar spectrum) of the Chinese ink-stained wood is 92.9% (Figure S15b).

As shown in Figure S15c, with increased water quantity, from 50 mL (corresponding to 2.0 cm in height) to 175 mL (7 cm in height), the evaporation rate of the 2D Chinese-ink stained wood with direct bulk water contact decreases dramatically from 1.12 kg m^{-2} h^{-1} to

0.83 kg m^{-2} h^{-1}. For comparison, the 3D CMF shows a stable evaporation rate of 3.15 kg with no dependence on water quantity (Figure S15d).

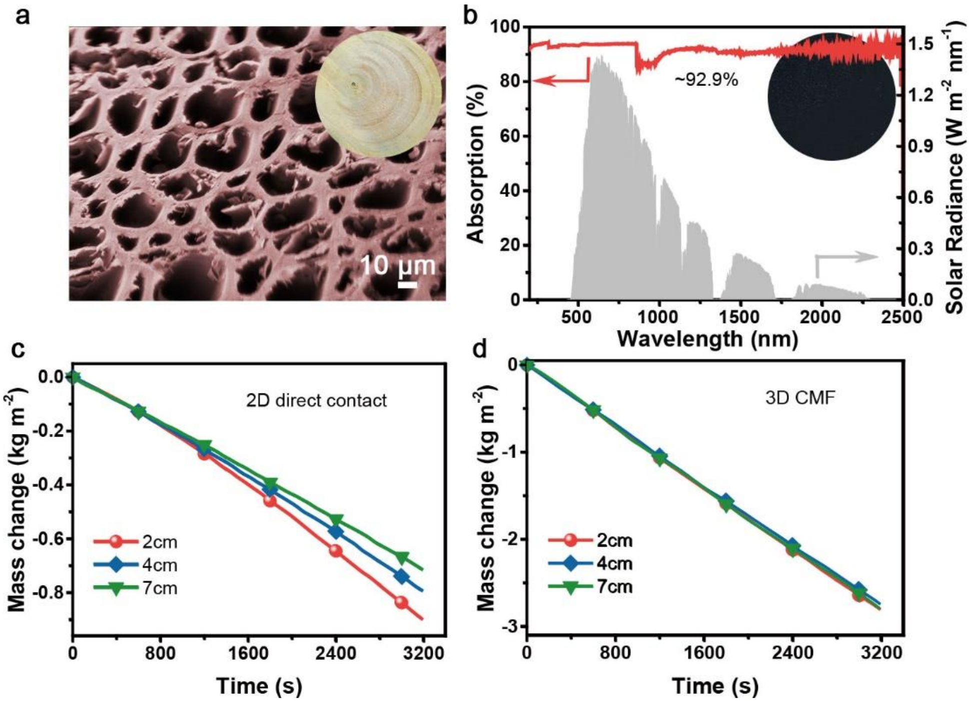

Figure S15. a) SEM image of the top view of the ginkgo wood. Inset shows the photograph of the block of the pristine wood. b) Absorption spectra of the Chinese ink-stained wood. Inset shows the photograph of the block of the wood after Chinese ink stained. c, d) Mass changes over time with 2D Chinese ink-stained wood (c) and the 3D CMF (d) using different water quantity.

S16 Dynamic Performance of 3D CMF and 2D Direct Contact.

In this experiment, discontinuous irradiation (in each cycle, there are 4 min of irradiation and 2 min without) was used to simulate changing cloud cover in real applications. Chinese ink-stained wood was selected a representative of 2D direct contact. As shown in **Figure S16**a, the ratio of mass change between discontinuous irradiation and continuous irradiation is about 70% for 2D direct contact, and the ratio is about 83.9% for 3D CMF (Figure S16b; Note: the Y coordinate is normalized). The result confirmed that as the 3D CMF based evaporator has a

shorter response time (less than 180 s), it will perform better in a real environment (under rolling cloud cover).

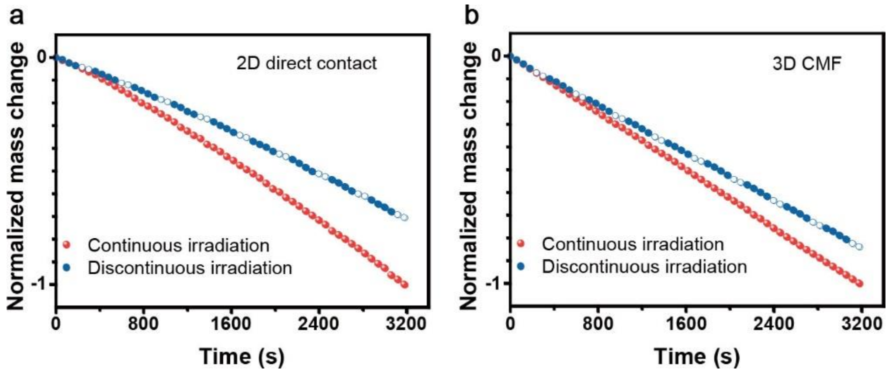

Figure S16. The performance of 2D direct contact (a) and 3D CMF (b) under continuous and discontinuous irradiation.

S17 Outdoor Experiment.

We performed outdoor experiments on the roof of the School of Electronic Science and Engineering at the Nanjing University Xian Lin campus in a typical cold winter day, during which the total solar flux reached a maximum value of 0.5 kW m^{-2}. Evidently, condensate water droplets evaporated by the 3D CMF can be observed on the glass cover of the balance (**Figure S17**c), while a little of condensate mist evaporated by the 2D Chinese ink-stained wood was observed after the same illumination time (Figure S17d), demonstrating the better evaporation performance of the 3D CMF.

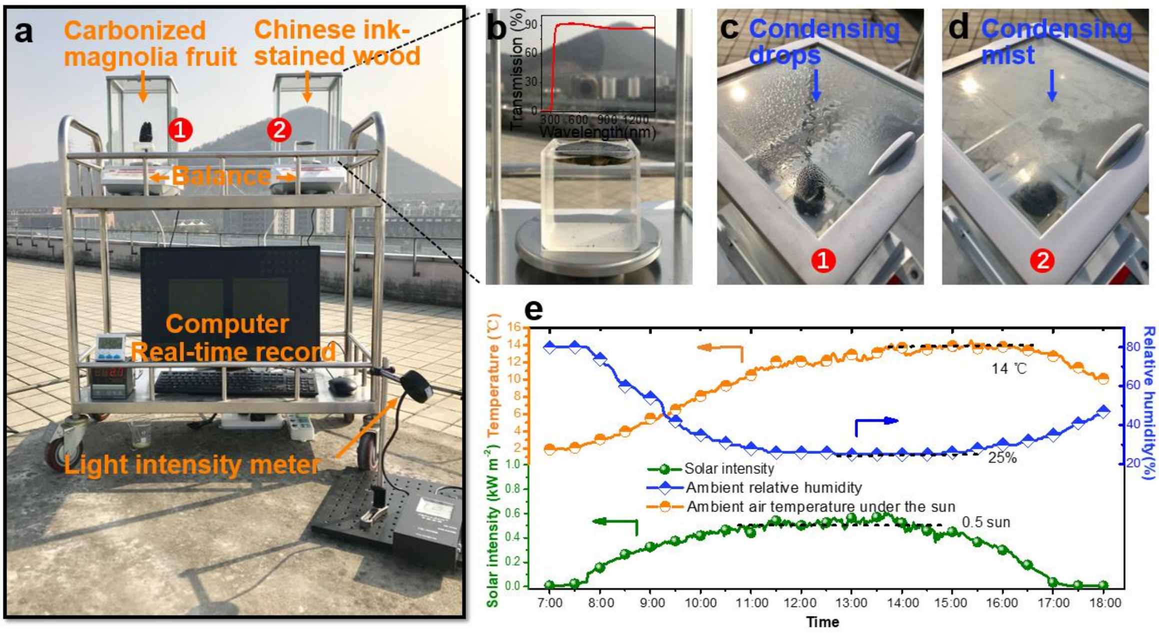

Figure S17. Rooftop experiments with the 3D CMF and Chinese ink stained wood under natural sunlight. a) Testing location on NJU's roof, in January 22, 2019. b) Photograph of the floating Chinese ink-stained wood. Inset shows the transmission curves for the glass cover of the balance. c, d) The photographs of condensate evaporated by the 3D CMF (c) and Chinese ink-stained wood (d) on the glass cover of the balance after the same irradiation time. e) The temperature, humidity and solar intensity over time.

S18 pH Independent Decontamination.

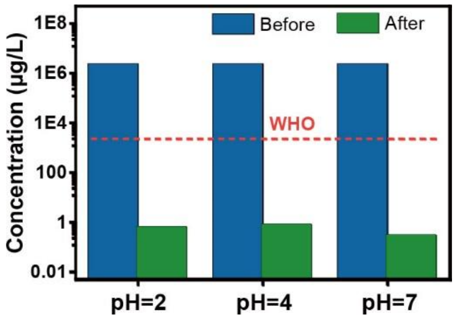

Figure S18. Concentrations of Cu^{2+} before and after treatment under different pH conditions. The dotted lines refer to the WHO standards for drinkable water.

S19 Long Time Durability Test for Wastewater Treatment.

We have performed the wastewater treatment experiment for 10 cycles, with 10 h illumination and 14 h in dark each cycle (Figure S19). Note that the starting ions' concentration (2 g/L) is at least 20 times higher than that of the actual plating wastewater (0.02~0.1 g/L). The durability test reveals that the CMF is quite stable for a long time.

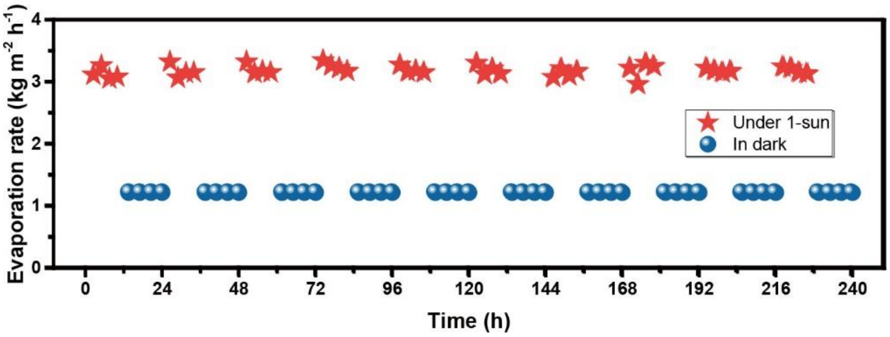

Figure S19. Stable cycling performance of the 3D CMF based evaporator for treating Cu^{2+}-contaminated water over 10 cycles. The stars and circles represent the evaporation rate at intervals of 2.5 h and 3.5 h, respectively.

S20 Mechanical Performance of 3D CMF.

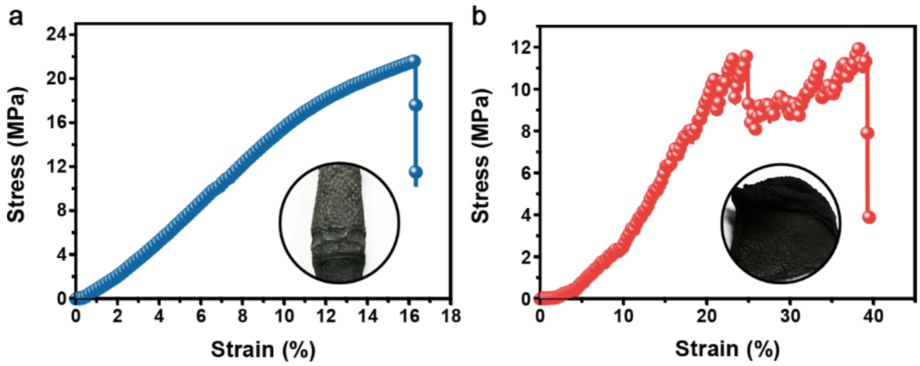

Figure S20. Flexural stress-strain curves of the pedicle (a) and carpel (b) of the CMF.

S21 Outstanding Corrosion Stability.

The CMF was immersed in concentrated H_2SO_4 (98%) and alkaline solution (PH=10, NaOH solution) for 1 month, respectively. Remarkably, the CMF exhibited excellent mechanical and chemical stability even under extreme environments.

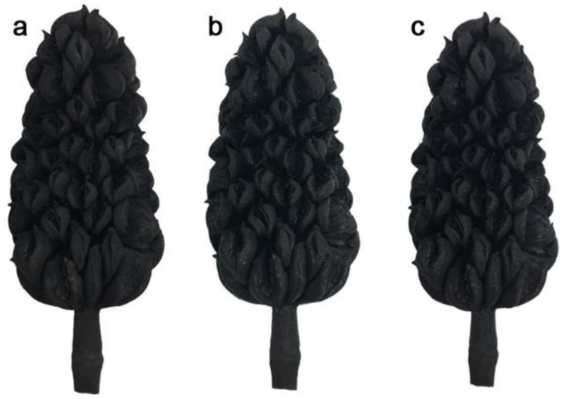

Figure S21. Photo images showing the stability of the CMF after soaked in acid (b) and alkaline solution (c) for 1 month, respectively.

S22 Carbonized magnolia fruit-based evaporator for freshwater production.

The 3D carbonized magnolia fruit solar-driven evaporation system was used for wastewater purification to produce freshwater (**Figure S22**a). A carbonized magnolia fruit was inserted into a piece of polystyrene foam with the thermal conductivity of ≈0.03-0.05 W m^{-1} k^{-1}, and the whole structure floated on the water surface with only the pedicle in contact with the simulated wastewater ($CuCl_2$ solution with the concentration of 200 g/L). The whole structure was put into a closed quartz container (Figure S22a). The evaporated water condensed on the transparent condenser (Figure S22b, c and d), and the water condensate flowed to the bottom of the prototype (Figure S22e). To determine the average rate of freshwater production, the set-up was illuminated under 1-sun for 11 h. Figure S22f shows the photos of the collected condensate. For comparison, the condensate evaporated without carbonized magnolia fruit was also collected. The average weight of the condensate generated by carbonized magnolia fruit is about 17.5 g. Considering the light absorption area is about 12.56 cm^2 (Diameter of the carbonized magnolia fruit: 4.0 cm), the average condensate collection rate is 1.27 kg m^{-2} h^{-1}, about 3.0 times that of evaporated without carbonized magnolia fruit (0.42 kg m^{-2} h^{-1}) (Figure S22g). Due to the reflection of the quartz cover (inset in Figure S22a), the scattering of the mist formed on the cover (Figure S22e) and the

$CuCl_2·5H_2O$ crystal accumulated on the evaporator surface (Figure 4f, in the manuscript), and the saturated internal humidity, the freshwater generation productivity is lower than the rate at the time of exposure (3.15 kg m^{-2} h^{-1}).

Note that the ion concentration of the actual plating wastewater (0.02~0.1 g/L) is at least 2000 times lower than the current experiment (200 g/L). Therefore, the average condensate collection rate of the CMF-based evaporator should be higher as the metal deposition is slow when applied to more general cases. Another vapor collection experiment was also carried out by using $CuCl_2$ solution with the concentration of 0.1 g/L to simulate the normal application situation. And the average condensate collection is 2.05 kg m^{-2} h^{-1}, 12 times higher than that of evaporated without carbonized magnolia fruit (0.154 kg m^{-2} h^{-1}) (Figure S22g).

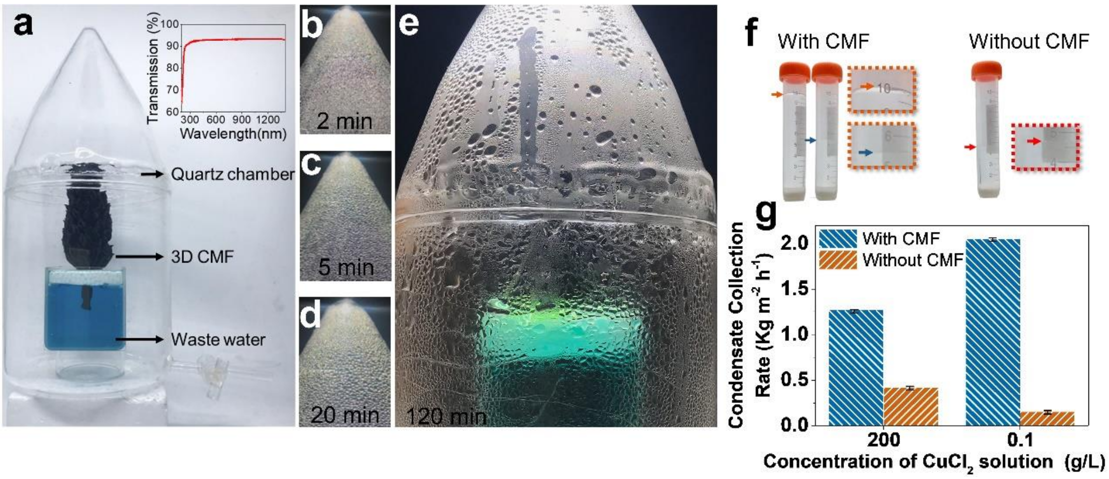

Figure S22. a) Digital photo of water collection device prototype based on 3D carbonized magnolia fruit. Inset shows the transmission curves for the quartz cover. b-e) The photographs of the quartz container during the experiment, showing (b) the obvious condensing mist in 2 minutes, (c, d) condensing drops and (e) rolling down of the condensing drops at the inner surface of the container. f) The photographs of the condensate evaporated with (left) and without (right) 3D carbonized magnolia fruit. Wastewater: 200 g/L $CuCl_2$ solution. g) Water collection performances with and without 3D carbonized magnolia fruit under 1 sun illumination.

Supplementary References

[1] V. Krassilov, S Barinova, *J. Plant Sci.* **2014,** 2, 282.

[2] F. Bonino, S. Brutti, P. Reale, B. Scrosati, L. Gherghel, J. Wu, K. Müllen, *Adv. Mater.* **2005,** 17, 743.

[3] J. Sodtipinta, C. Ieosakulrat, N. Poonyayant, P. Kidkhunthod, N. Chanlek, T. Amornsakchai, P. Pakawatpanurut, *Ind. Crops Prod.* **2017,** 104, 13.

[4] B. Y. Liu, R. C. Jordan, *Sol. Energy* **1960,** 4, 1.

[5] M. A. Green, Solar cells: operating principles, technology, and system applications. **1982.**

[6] G. Ni, G. Li, S. V. Boriskina, H. X. Li, W. L. Yang, T. J. Zhang, G. Chen, *Nat. Energy* **2016,** 1, 16126.

[7] R. W. Boyd, *Am. J. Phys.* **1984,** 52, 668.

[8] M. Badioli, A. Woessner, K. J. Tielrooij, S. Nanot, G. Navickaite, T. Stauber, F. J. García de Abajo, F. H. Koppens, *Nano Lett.* **2014,** 14, 6374.

[9] P. L. Ong, W. B. Euler, I. A. Levitsky, *Appl. Phys. Lett.* **2010,** 96, 033106.

[10] B. Bellich, M. Borgogna, M. Cok and A. Cesàro, *J. Therm. Anal. Calorim.* **2010,** 103, 81.

[11] H. C. Yang, Z. Chen, Y. Xie, J. Wang, J. W. Elam, W. Li, S. B. Darling, *Adv. Mater. Interfaces* **2019,** 6, 1801252.

[12] X. F. Huang, Q. Wang, X. X. Liu, S. H. Yang, C. X. Li, G. Sun, L. Q. Pan, K. Q. Lu, *J. Phys. Chem. C* **2019**, 113, 18768.

[13] H. C. Chen, H. C. Lin, H. H. Chen, F. D. Mai, Y. C. Liu, C. M. Lin, C. C. Chang, H. Y. Tsai, C. P. Yang, *Sci. Rep.* **2014,** 4, 4425.

[14] F. Zhao, X. Zhou, Y. Shi, X. Qian, M. Alexander, X. Zhao, S. Mendez, R. Yang, L. Qu,

G. Yu, *Nat. Nanotechnol.* **2018,** 13, 489.

[15] K. Kudo, J. Ishida, G. Syuu, Y. Sekine, T. Ikeda-Fukazawa, *J. Chem. Phys.* **2014,** 140, 044909.

[16] K. Kudo, J. Ishida, G. Syuu, Y. Sekine, T. Ikeda-Fukazawa, *J. Chem. Phys.* **2014,** 140, 044909.

[17] C. A. Hassan, B. Francis, F. Linda, M. Mathieu, C. Florent, F. Valérian, F. Bernadette, F. Michel, D. M. Tilmann, *Angew. Chem. Int. Ed.* **2015,** 54, 14685.

[18] X. Li, Z. Z. Yang, *J. Phys. Chem.* **2005,** 109, 4102.

[19] D. E. Bacelo, Y. Ishikawa, *Chem. Phys. Lett.* **2000,** 319, 679.

[20] Y. Yang, R. Zhao, T. Zhang, K. Zhao, P. Xiao, Y. Ma, P. M. Ajayan, G. Shi, Y. Chen, *ACS Nano* **2018,** 12, 829.

[21] F. Liu, B. Zhao, W. Wu, H. Yang, Y. Ning, Y. Lai, R. Bradley, *Adv. Funct. Mater.* **2018,** 28, 1803266.

[22] Y. Yang, W. Que, J. Zhao, Y. Han, M. Ju, X. Yin, *Chem. Eng. J.* **2019,** 373, 955.

[23] Y. Shao, Z. Jiang, Y. Zhang, T. Wang, P. Zhao, Z. Zhang, J. Yuan, H. Wang, *ACS Nano* **2018,** 12, 11704.

[24] M. Gao, C. K. Peh, H. T. Phan, L. Zhu, G. W. Ho, *Adv. Energy Mater.* **2018,** 8, 1800711.

[25] L. Zhu, L. Sun, H. Zhang, D. Yu, H. Aslan, J. Zhao, Z. Li, M. Yu, F. Besenbacher, Y. Sun, *Nano Energy* **2019,** 57, 842.

[26] X. Ma, W. Fang, Y. Guo, Z. Li, D. Chen, W. Ying, Z. Xu, C. Gao, X. Peng, *Small* **2019,** 15, 00354.

[27] G. Zhang, Z. Duan, X. Qi, Y. Xu, L. Li, W. Ma, H. Zhang, C. Liu, W. Yao, *Carbon* **2019,** 148, 1.

[28] W. Xu, Y. Xing, J. Liu, H. Wu, Y. Cui, D. Li, D. Guo, C. Li, A. Liu, H. Bai, *ACS Nano* **2019,** 13, 7930.

[29] J. Yang, Y. Pang, W. Huang, S. K. Shaw, J. Schiffbauer, M. A. Pillers, X. Mu, S. Luo, T. Zhang, Y. Huang, G. Li, S. Ptasinska, M. Lieberman, T. Luo, *ACS Nano* **2017,** 11, 5510.

[30] Y. Fu, G. Wang, T. Mei, J. Li, J. Wang, X. Wang, *ACS Sustainable Chem. Eng.* **2017,** 5, 4665.

[31] Q. Jiang, L. Tian, K. K. Liu, S. Tadepalli, R. Raliya, P. Biswas, R. R. Naik, S. Singamaneni, *Adv. Mater.* **2016,** 28, 9400.

[32] J. Wang, Z. Liu, X. Dong, C. E. Hsiung, Y. Zhu, L. Liu, Y. Han, *J. Mater. Chem. A* **2017,** 5, 6860.

[33] Y. Ito, Y. Tanabe, J. Han, T. Fujita, K. Tanigaki, M. Chen, *Adv. Mater.* **2015,** 27, 4302.

[34] Y. Fu, G. Wang, X. Ming, X. Liu, B. Hou, T. Mei, J. Li, J. Wang, X. Wang, *Carbon* **2018,** 130, 250.

[35] K. Kim, S. Yu, C. An, S. W. Kim, J. H. Jang, *ACS Appl. Mater. Interfaces* **2018,** 10, 15602.

[36] W. Zhang, W. Zhu, S. Shi, N. Hu, Y. Suo, J. Wang, *J. Mater. Chem. A* **2018,** 6, 16220.

[37] F. Zhang, Y. Li, X. Bai, S. Wang, B. Liang, G. Fu, Z. S. Wu, *J. Mater. Chem. A* **2018,** 6, 23263.

[38] P. Mu, Z. Zhang, W. Bai, J. He, H. Sun, Z. Zhu, W. Liang, A. Li, *Adv. Energy, Mater.* **2018,** 9, 1802158.

[39] P. Qiu, F. Liu, C. Xu, H. Chen, F. Jiang, Y. Li, Z. Guo, *J. Mater. Chem. A* **2019,** 7, 13036.

[40] X. Y. Wang, J. Xue, C. Ma, T. He, H. Qian, B. Wang, J. Liu, Y. Lu, *J. Mater. Chem. A* **2019,** 7, 16696.

[41] G. Zhu, J. Xu, W. Zhao, F. Huang, *ACS. Appl. Mater. Interfaces* **2016,** 8, 31716.

[42] M. Kaur, S. Ishii, S. L. Shinde, T. Nagao, *ACS Sustainable Chem. Eng.* **2017,** 5, 8523.
